# Cardiac Remodeling in Cancer-Induced Cachexia: Functional, Structural, and Metabolic Contributors

**DOI:** 10.3390/cells11121931

**Published:** 2022-06-15

**Authors:** Michael P. Wiggs, Anna G. Beaudry, Michelle L. Law

**Affiliations:** 1 Department of Health, Human Performance, and Recreation, Robbins College of Health and Human Sciences, Baylor University, Waco, TX 76706, USA; michael_wiggs@baylor.edu (M.P.W.); annagrace_beaudry1@baylor.edu (A.G.B.); 2 Department of Human Sciences and Design, Robbins College of Health and Human Sciences, Baylor University, Waco, TX 76706, USA

**Keywords:** cancer cachexia, cardiomyopathy, cardiac atrophy, systolic, diastolic, heart, mitochondria, inflammation, oxidative stress

## Abstract

Cancer cachexia is a syndrome of progressive weight loss and muscle wasting occurring in many advanced cancer patients. Cachexia significantly impairs quality of life and increases mortality. Cardiac atrophy and dysfunction have been observed in patients with cachexia, which may contribute to cachexia pathophysiology. However, relative to skeletal muscle, little research has been carried out to understand the mechanisms of cardiomyopathy in cachexia. Here, we review what is known clinically about the cardiac changes occurring in cachexia, followed by further discussion of underlying physiological and molecular mechanisms contributing to cachexia-induced cardiomyopathy. Impaired cardiac contractility and relaxation may be explained by a complex interplay of significant heart muscle atrophy and metabolic remodeling, including mitochondrial dysfunction. Because cardiac muscle has fundamental differences compared to skeletal muscle, understanding cardiac-specific effects of cachexia may bring light to unique therapeutic targets and ultimately improve clinical management for patients with cancer cachexia.

## 1. Introduction

### 1.1. Cancer Cachexia Overview

Cancer cachexia is a multifactorial syndrome of progressive and unintentional weight loss. It occurs in 30% of all cancer patients and 70–90% of patients with cancers of the lung, liver, and gastrointestinal tract [1]. This condition is thought to contribute to ~20% of cancer deaths [2]. Prevalence statistics for cancer cachexia are not included in the national cancer records of any country [3], so these suggested percentages may underestimate the true prevalence. Cancer is a leading cause of death worldwide, accounting for nearly 10 million deaths in 2020 [4]. Many of these cancer deaths result from cancers commonly associated with cachexia, such as gastric, pulmonary, pancreatic, esophageal, hepatic, and colorectal cancers [5].

An international Delphi consensus definition and diagnostic classification for cancer cachexia was published in 2011 [6]. As outlined in this document, a cancer cachexia diagnosis should be made when body weight loss is greater than 5% within six months, when body mass index (BMI) is less than 20 kg/m^2^ in combination with body weight loss of greater than 2%, or when appendicular skeletal muscle index is consistent with sarcopenia and weight loss is greater than 2% [6]. These are the current diagnostic criteria; however, uncertainty remains as to whether more specificity may be beneficial for purposes of identification and prevention. Specifically, body composition (fat vs. lean mass) and changes in body composition over time may be important predictors of morbidity and mortality [7,8]. The discovery of specific biomarkers that predict for cancer-induced weight loss would also be diagnostically advantageous [9].

In this multifactorial syndrome, there is ongoing muscle and adipose wasting, leading to progressive functional decline. Patients often exhibit reduced tolerance and effectiveness of cancer therapies [10], such as radiotherapy and chemotherapy, as well as increased risk of chemotoxicity. In addition to increased mortality, cancer cachexia causes profound fatigue and weakness, decreases quality of life [11], increases the occurrence of cancer-related complications [12], and increases medical costs [13]. Although anamorelin–a ghrelin receptor agonist–was recently approved to treat cachexia in Japan [14], therapeutic options remain very limited. Furthermore, because of the complexity of cachexia pathology, a multimodal treatment strategy is likely required to adequately address the multiple facets of this syndrome [15].

Cancer cachexia can be described as a condition involving systemic inflammation, metabolic dysregulation, and decreased food intake leading to energy imbalance [3]. Unlike starvation, cancer cachexia cannot be completely reversed by adequate nutrition alone [16,17]. Resting energy expenditure is often elevated—at least in part due to tumor metabolism [18,19,20], inflammation [21,22,23], mitochondrial dysfunction [24], and futile cycling [25,26,27]—thus, promoting a negative energy balance [28,29]. Abnormalities in protein synthesis and degradation, as well as amino acid transport, are observed in cachectic skeletal muscle. Proteolysis is highly upregulated while the rate of protein synthesis declines [30,31]. Muscle breakdown during cancer cachexia occurs with significant involvement of the ubiquitin proteasome system (UPS) and autophagy lysosome system (ALS) [32]. There is also evidence of increased apoptosis, impaired muscle regenerative capacity [33], and increased branched-chain amino acid oxidation [30]. In combination, these alterations lead to the highly catabolic state that is characteristic of cancer-induced cachexia. Evidence suggests that other tissues and organs, including heart, adipose, brain, liver, bone, blood, and gut, are also adversely affected by cachexia and may contribute to cachexia pathology [30,34]. These systemic implications are thought to be caused by inflammatory factors released from both tumor and host tissues.

### 1.2. Cancer Cachexia and the Heart

**Figure 1:**
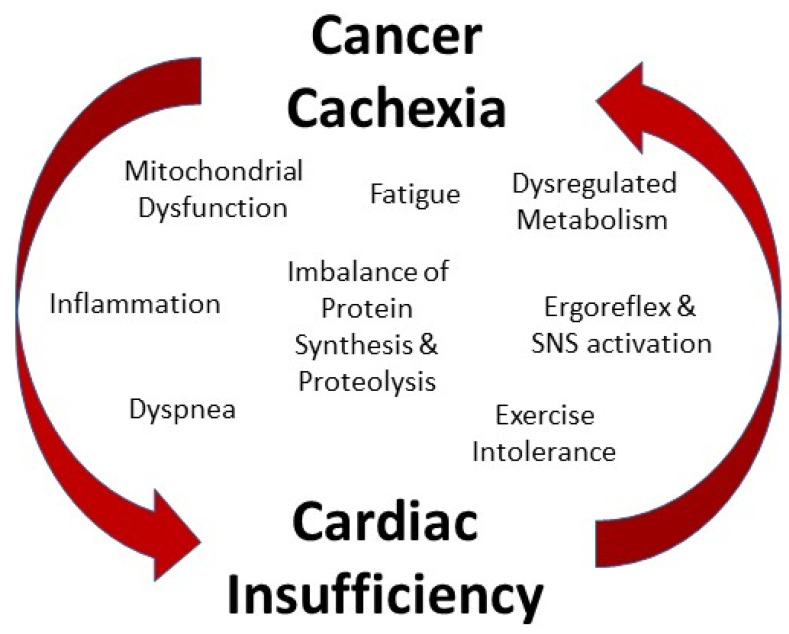
Interrelationship between cancer cachexia and cardiac insufficiency is found in multiple common symptoms and mechanistic contributors. SNS (sympathetic nervous system).

In addition to cancer cachexia’s significant deleterious effects on skeletal muscle size and function, increasing evidence points to adverse effects of cachexia on cardiac muscle as well. However, relative to skeletal muscle, little work has been done clinically to identify effects of cancer cachexia on the heart or experimentally to uncover underlying mechanisms driving cachexia-induced cardiac functional changes. Nevertheless, the importance of understanding cardiac pathology in cancer cachexia is emphasized by the fact that cachexia is also present in a high percentage of heart failure patients [35], uncovering a potential link between cardiac disease and skeletal muscle wasting. Due to many similar mediating factors between cancer cachexia and heart failure, it is plausible that cancer-induced cachexia may contribute to cardiac dysfunction, and cardiac dysfunction may in turn exacerbate cachexia to create a vicious cycle contributing to morbidity and mortality [36] (Figure 1).

The increasing emphasis in recent years on evaluating and treating heart disease in cancer patients has been driven in large part by the known cardiotoxicity of many chemotherapeutic agents as well as pre-existing cardiovascular disease in many cancer patients [37,38]. These factors have led to growth in the field of cardio-oncology [39]. Furthermore, improvements in early cancer detection and development of more targeted and effective cancer therapies are leading to remission or stabilized disease with increased length of life, even for some patients with advanced disease. As a result, more cancer patients die of causes not directly related to tumor burden. According to the National Cancer Institute’s Surveillance, Epidemiology, and End Results (SEER) data, deaths from cancer among patients who have had a cancer diagnosis have progressively declined from approximately 80% to 60% over a 40-year period from 1973 to 2012, and deaths from other causes have increased [40]. Heart disease is the greatest cause of non-cancer death among cancer patients, accounting for approximately half of all non-cancer deaths [40]. The risk of heart-related death is 2-fold higher in patients who have had a cancer diagnosis compared to the general population [40]. Among patients actively being treated for cancer, two different studies found that ~7% (35/506 patients) [41] and 9% (75/816 patients) [42] died of cardiac failure or acute myocardial infarction and not directly from the tumor.

To date, the field of cardio-oncology has not focused largely on cachexia as a contributor to cardiac disease in cancer patients. Indeed, teasing apart individual effects of cancer therapies, pre-existing heart disease, and cachexia in a clinical setting is difficult in many situations. However, both clinical and pre-clinical evidence of direct effects of cachexia on the heart is growing. The purpose of this review is to provide a discussion of what has been observed clinically and what is known mechanistically related to the effects of cachexia on cardiac (1) functional remodeling, (2) structural remodeling, and (3) metabolic remodeling, and what questions remain. An understanding of cancer cachexia-induced cardiac pathophysiology is imperative to developing optimal treatment plans and therapeutic strategies for cancer patients. As cardiac muscle has minimal regenerative capacity [43], protecting the heart is essential to increasing both quality and length of life for individuals with cancer diagnoses.

## 2. Functional Remodeling

Evidence of impaired cardiac function in cancer-induced cachexia has grown in recent years. Importantly, cardiac functional deficits may directly contribute to cachexia-related fatigue, consequently diminishing whole body functional capacity and quality of life. The following section reviews what is known about cardiac function in patients with cachexia and follows with physiological underpinnings in pre-clinical models.

### 2.1. Clinical Observations of Cardiac Functional Changes

Elevated heart rate has been observed in multiple clinical studies of cancer patients, both with and without overt cachexia. Heart rate elevation was present across multiple types of cancer, including breast [44], colon [45,46], lung [45], and pancreatic [45], in both treatment-naïve [45,46] and chemotherapy treated groups [45]. Importantly, elevated heart rate was associated with increased mortality [44,45]. Increased sympathetic nervous system (SNS) activation is a likely contributor to increased chronotropy [47]. Decreased heart rate variability [46] and increased serum B-type natriuretic peptide (BNP) [48] also provide evidence of impaired sympathoadrenal signaling in cachectic patients. Impaired oxygen uptake (VO_2_ max) [46] and anemia [49] may have a role in SNS activation in cachexia. Moreover, increased ergoreflex activation due to metabolic and structural changes in skeletal muscle has also been implicated in increased SNS activity [50,51], although the specific effects of ergoreflex activation in cancer-induced cachexia have not been studied to our knowledge.

It was hypothesized that cachexia may increase susceptibility to electrical abnormalities in the hearts of cachectic patients as a result of cardiac atrophy and increased ventricular wall stress [52]. Electrocardiogram (ECG) measurements, including PQ, QRS, and QTc duration, were not different between cancer patients and controls when data were collected at a single time point [45]. However, when a subset of these patients underwent 24-hour ECG testing, 9/120 cancer patients and 0/43 control patients had ≥1 episodes of non-sustained ventricular tachycardia, which predicted for increased mortality after 5 years [53]. Although this study did not specifically look at cachexia, colorectal, lung, and pancreatic cancer patients included in this cohort have a high incidence of cancer-induced weight loss [1].

Cardiac contractile function in cancer patients has also been measured by echocardiography studies. Treatment-naïve colorectal cancer (CRC) patients had decreased left ventricular ejection fraction (LVEF)–a measure of systolic function–compared to control subjects at a single time point [46]. Stage IV non-small cell lung cancer (NSCLC) patients underwent echocardiography before and ~4 months following carboplatin therapy. LVEF and global longitudinal strain (GLS), another measure of systolic function, were significantly decreased at 4 months compared to baseline [54]. The effects of carboplatin versus cancer on cardiac function cannot be determined from this study. Carboplatin is in the class of drugs known as alkylating agents, some of which may have cardiotoxic effects [55]. However, loss of left ventricular mass was associated with the largest decrease in GLS [54], suggesting an association between functional decline and cardiac atrophy. It is important to note, despite significant differences in LVEF in the former two studies, neither study reported mean LVEF values that would be considered clinically abnormal (< 50%). A study in treatment-naïve NSCLC patients without a history of cardiovascular disease did identify 7/70 patients with LVEF < 50%. Of these seven patients, five were identified as cachectic and one was identified as pre-cachectic [56]. Because cardiac dysfunction is also a risk factor for the development of cachexia [35], the causal direction between cachexia and systolic dysfunction is unknown. However, this study identifies a significant association between cachexia and cardiac abnormalities, which complicates the clinical picture for this subset of cancer patients. A summary of clinical studies identifying functional cardiac remodeling is found in Table 1.

### 2.2. Mechanistic Contributors to Cardiac Functional Changes

Echocardiographic abnormalities have also been observed in multiple pre-clinical models of cachexia, providing clinical relevance to these models. Colon-26 Adenocarcinoma (C26) [61,62,63,64,65,66,67,68,69], Yoshida AH-130 Ascites [48,70,71], and Ehrlich Ascites Carcinoma [72] models all show diminished in vivo cardiac performance. Cardiac function in the Lewis Lung Carcinoma (LLC) model appears to be sex-specific, with males having impaired function and females showing protection against tumor-induced functional changes [69,73]. Most studies identify systolic functional deficits measured by decreased fractional shortening (FS) or percent ejection fraction (%EF) [48,61,62,64,65,67,68,69,70,71,72], although stroke volume [62,71] and cardiac output [48,70] are also reported. Fewer studies have assessed in vivo diastolic performance. Xu et al. reported no difference in the E/A ratio [65]; E/A is the ratio of early to late ventricle filling. Diastolic dysfunction is characterized by a non-compliant heart, leading to less early filling and a lower E/A ratio. Tei index, a measure of global systolic and diastolic left ventricle function, was unchanged in one study [65] and increased (indicating worsened function) in another [68]. Despite little difference in diastolic function in echocardiography studies, invasive in vivo hemodynamics measurements identified significantly impaired diastolic function–measured by left ventricular end-diastolic pressure (LVEDP) and negative pressure derivative (-dP/dt; relaxation rate) in the Yoshida AH-130 rat cachexia model [48]. The increased resting heart rate that has been noted in clinical studies of cachectic patients has not been recapitulated in animal models. Heart rate in pre-clinical echocardiography studies has either been unchanged [64,65,73] or decreased [48,67]. However, telemetry studies may provide a more accurate understanding of heart rate fluctuations and heart rate variability in animal models as echocardiography in rodents must be carried out under anesthesia.

Underlying physiological contributors to impaired in vivo function have been examined in ex vivo isolated hearts to identify functional aberrations outside of the diseased animal [74]. In contrast to in vivo studies, working heart preparations from tumor-bearing cachectic rats unexpectedly had increased baseline function measured by coronary flow, cardiac output, and oxygen consumption [75,76]. Hearts from tumor-bearing rats also had increased β-adrenergic sensitivity, evidenced by a greater increase in heart rate, peak systolic pressure, and positive pressure derivative (+dP/dt; contraction rate) in response to isoproterenol treatment, compared to control hearts [75,76]. In a similar experiment using the C26 mouse model of cachexia, Law and Metzger found Langendorff-perfused hearts had decreased left ventricular developed pressure (LVDP; peak pressure) and +dP/dt, both indicating decreased systolic function [74]. Furthermore, significant impairment in diastolic function was uncovered. Cachectic hearts had diminished -dP/dt and increased LVEDP under pacing stress conditions, indicating incomplete relaxation at higher heart rates [74]. The reason for the discrepancy in findings between Drott et al. [75,76] and Law and Metzger [74] are unclear, although model-specific differences may exist. Additionally, there are differences between the Langendorff and working heart preparations. Langendorff involves retrograde perfusion through the aorta, with a water-filled balloon inserted into the left-ventricle to measure pressure. The heart is paced via electrical stimulation and is thus controlled. Working heart preparations mimic in vivo blood flow, with perfusate entering the left atrium via the pulmonary vein. Hearts are not paced, and the heart pumps perfusate out of the aorta against a resistance [77]. Regardless of the differences in outcome, these experiments are significant as they establish cardiac-intrinsic functional changes between control and tumor-bearing animals under controlled experimental conditions, providing hearts with equal oxygenation and energy substrate availability. More work is needed to identify the mechanistic underpinnings of the differential results between different models and heart preparations.

Changes in isolated cardiac myocyte function have also been uncovered in the C26 model of cachexia, although, similar to ex vivo heart function, findings between studies are somewhat inconsistent. Law and Metzger [74] found a significantly decreased peak height of myocyte contraction, and a slowed rate of contraction and relaxation. Underlying these functional deficits was a profound decrease in calcium cycling. Decreased peak calcium and increased time to peak calcium and 50% calcium decay closely mirrored the deficits in contraction and relaxation, uncovering an important cellular-intrinsic physiological mechanism of impaired contractility and relaxation. Of note, three other studies using the C26 model found decreased in vivo contractility (%EF and FS), but this did not correlate with changes in the peak height of contraction in isolated myocytes. Furthermore, the studies had mixed results regarding myocyte relaxation and changes in calcium cycling [61,62,65]. Two major differences exist between these studies. The former study [74] used male mice that had undergone significant body wasting (~20% difference in body weight between tumor and control groups) and cardiac atrophy (14% difference between tumor and control groups). Female mice with less severe body wasting and no difference in cardiac mass were used in the latter studies [61,62,65]. Importantly, the differences between these studies suggest a role for cachexia severity in cardiac functional deficits, and also reveals potential sex differences between the onset and progression of cardiac dysfunction in cancer-induced cachexia. The time course of cardiomyopathy development and sex differences in cardiac dysfunction in cachexia deserve further investigation.

In conclusion, significant evidence points to in vivo contractility deficits in experimental cachexia. The isolated heart and cardiac myocyte experiments discussed here provide insight into the physiological underpinnings of in vivo cardiac dysfunction. Although findings differ based on the model, functional assay, sex, and cachexia severity, it is evident that heart- and myocyte-intrinsic alterations in cardiac function exist outside of the diseased animal. The molecular mechanisms of these physiological changes are likely complex and multifactorial. Changes in contractility and calcium cycling in cardiac myocytes likely stem from tumor-induced systemic changes including inflammation, altered substrate availability, and changes in sympathoadrenal signaling. These extrinsic signals lead to intrinsic changes in gene and protein expression and post-translational modifications of proteins within the myocyte, converging to induce structural and metabolic remodeling which ultimately affects cardiac performance. Structural and metabolic changes in the heart during cachexia are discussed in the subsequent sections.

## 3. Structural Remodeling

Numerous organ- and cellular-level structural changes occur in the heart muscle during cachexia development, which may contribute to impaired cardiac function. Most notably, profound atrophy of the heart muscle is found in humans and animal models of cachexia. Underlying the atrophic phenotype are cellular-level changes in sarcomere protein expression and rates of protein synthesis and degradation.

### 3.1. Clinical Observations of Cardiac Structural Changes

In 1968, Burch et al. described a condition termed the “cachectic heart” that occurred in patients with malignancy and other chronic conditions resulting in malnutrition or inactivity. The cachectic heart was defined as “an acquired pathologic decrease in size, mass, and [epicardial] fat content of the heart with little or no atherosclerosis of the aorta and coronary arteries” [78]. Other observable anatomical and structural changes included lymphocyte infiltration, a flabby appearance of the ventricles, spaces between atrophied muscle fibers, absence of ischemic disease, and an estimated loss of cardiac mass approximately proportional to the total loss of body mass [78]. These morphological changes are in stark contrast to “classic” heart failure, which is characterized by a compensatory hypertrophic remodeling phase, followed by pump failure with dilation and thinning of the ventricular walls [79]. More recent studies have confirmed Burch’s findings of a profound decrease in cardiac mass in cancer patients. In general, an increase in total body weight loss is associated with increased atrophy of the cardiac muscle. Examination of post-mortem cardiac mass in patients with cancer cachexia, cancer patients without cachexia, and patients dying from other causes revealed significantly reduced total cardiac mass and left-ventricle wall thickness with increased fibrosis in cachectic patients compared to both non-cachectic cancer patients and control patients [48]. A retrospective study of 177 patients who died of lung, gastrointestinal, and pancreatic cancer and without history of cardiovascular disease confirmed these findings, also identifying significantly decreased cardiac mass in cachectic patients [57]. However, cachectic patients in this study also had higher rates of chemotherapy and radiotherapy compared to non-cachectic patients, making cachexia-specific effects on cardiac size difficult to ascertain. Nevertheless, heart mass was correlated to body weight in all patients, suggesting body weight loss alone may have some effect on cardiac atrophy [57].

Cardiac atrophy in cancer patients is associated with worsened clinical outcomes. In treatment-naïve advanced pancreatic cancer patients, a decreased left ventricular muscle area (LVMA) predicted shortened overall survival, and left ventricular muscle radiation attenuation (LVMRA) predicted a reduction in both overall and progression-free survival [58]. Muscle radiation attenuation measures muscle density via computed tomography (CT) scan, with lower attenuation scores indicating intramuscular fat deposition. In skeletal muscle, decreased muscle attenuation is associated with decreased strength, independent of total muscle mass [80]. A potential limitation of this study is the relatively infrequent use of CT scans to measure cardiac mass, but an echocardiography study by Kazemi-Bajestani et al. is in agreement with these findings. Kazemi-Bajestani et al. conducted a prospective, longitudinal study of 50 NSCLC patients. Cardiac tissue atrophy over time, measured by echocardiography, was a predictor of disease severity and clinical outcomes [54]. This study included cardiac tissue mass along with skeletal muscle and adipose mass in the analyses. As the number of tissues exhibiting atrophy increased, there was a decrease in physical functioning, chemotherapy response, and survival. Moreover, cardiac atrophy was independently associated with dose-limiting toxicity of chemotherapy [54]. A summary of clinical studies identifying structural remodeling is found in Table 1.

### 3.2. Mechanistic Contributors to Cardiac Structural Changes

Cardiac atrophy is also noted in multiple pre-clinical models of cancer cachexia [48,67,81], providing consistency between findings in animals and humans with cachexia. Decreased ventricular mass in animals has been attributed to decreased thickness of the interventricular septum [66] and left ventricle posterior wall [66,71] without an increase in left ventricular diameter at diastole [66,71]. These findings, together with decreased cardiac myocyte width in the absence of increased length [74], suggest an atrophic rather than a dilated phenotype, consistent with clinical observations. Underlying cardiac atrophy is an alteration in the expression of myofilament proteins, specifically a decreased total protein expression of myosin heavy chain (MHC) [48,66] and troponin I (cTnI) [66] in whole heart homogenates. A proteomics study in hearts of C26 tumor-bearing mice found an increase in multiple sarcomeric proteins in the soluble protein fraction, including multiple isoforms of MHC, actin, tropomyosin, and troponin, as well as titin. The authors suggested the increase was due to a destabilized sarcomere and increased release of sarcomeric proteins for degradation [82]. Indeed, sarcomeric ultrastructural changes identified via electron microscopy indicate disorganized and destabilized sarcomeres [67,82], which could significantly impair contractile function within the cardiac muscle.

In addition to total loss of sarcomeric proteins and sarcomere ultrastructural disarray, increased fibrosis and a decreased ratio of α/β-MHC may also have a role in impaired cardiac function in cachexia. Fibrosis, associated with a noncompliant heart that impedes relaxation and diastolic function [83], has been found in multiple cachexia studies [64,67,74,84]. Although significant fibrosis likely contributes to impaired diastolic function in cachexia, fibrosis-independent mechanisms are also involved, as isolated cardiac myocytes freed from fibrotic lesions also exhibit slow relaxation properties [65,74]. Fetal gene reactivation in heart failure is associated with a decrease in the “fast” α-MHC isoform and an increase in the “slow” β-MHC isoform. Both mRNA and protein analyses reveal a decreased ratio of α/β-MHC in hearts of C26 tumor-bearing mice [66,67], similar to other causes of heart failure in humans [85,86]. β-MHC exhibits slower cross-bridge kinetics, decreasing maximum shortening velocity and peak contraction compared with α-MHC. Interestingly, the change in contractility resulting from the MHC isoform switch is calcium independent [87,88], suggesting that both calcium-dependent [74] and calcium-independent mechanisms may be contributing to impaired contractile function.

Muscle size, including cardiac muscle, is controlled by the relative rates of protein synthesis and degradation. Upregulation of UPS, ALP, and apoptosis are known to promote cancer cachexia [89,90]. The hyperactivity of these pathways leads to muscle atrophy. Various pre-clinical studies exploring cancer-induced cardiac muscle wasting are at odds regarding which proteolytic pathway is predominant in the heart during cancer cachexia. Some work suggests that upregulation of the UPS and apoptosis/caspase proteolytic systems may be involved in the loss of myofibrillar protein in cachectic hearts [48,65,66,91,92]. Others deem cardiac muscle wasting the result of increased activation of the ALP [71,84,93]. It is possible that alterations to any of these three pathways may lead to cardiac muscle wasting in the cachectic heart, and different mechanisms of proteolysis may be predominant at different stages of cachexia and with different models of cachexia. In addition to increased proteolysis, a decrease in protein synthesis also contributes to the net loss of cardiac muscle protein in cachexia. The protein synthesis rate in the hearts of C57BL/6 mice with Apc^Min/+^ induced cancer cachexia was found to be reduced by approximately 70% compared to control mice [93]. Similarly, cardiac protein synthesis was reduced in LLC tumor-bearing male mice compared to control mice [69]. Taken together, both increased protein degradation and decreased protein synthesis have been observed in the hearts of cachectic animals. Thus, net protein balance in the cachectic heart disproportionately favors catabolism.

## 4. Metabolic Remodeling

Metabolic changes, including inefficient energy production, increased metabolic rate, and dysregulation of metabolic pathways, are paramount in the development and progression of cachexia-induced skeletal muscle wasting. Underlying many of these changes is a profound decrease in the function of mitochondria, which are responsible for approximately 90% of the body’s energy production. Due to the high energetic requirements of cardiac muscle, it seems reasonable to suggest that metabolic aberrations in cachexia likely have an important role in the pathogenesis of cachexia-induced cardiomyopathy as well.

### 4.1. Clinical Observations of Cardiac Metabolic Changes

Clinically, little is known about changes in cardiac metabolism in cancer cachexia, but limited evidence suggests an increased energy expenditure and altered substrate utilization in cardiac tissue. Increased resting heart rate has been noted in cachectic patients, and although not necessarily causal, this was significantly correlated with body weight-adjusted resting energy expenditure [94]. Indeed, cardiac energy expenditure accounts for approximately 10% of resting metabolic rate in healthy adults [95], meaning increased heart rate may well contribute to increased whole body energy utilization. Treatment of weight-losing cancer patients with propranolol (beta-adrenergic receptor antagonist) led to a decrease in resting energy expenditure compared to treatment with indomethacin (prostaglandin synthesis inhibition), morphine (pain reliever), or placebo, implicating increased sympathetic nervous system activity as a contributor to increased energy expenditure in weight-losing cancer [59]. In a crossover study, atenolol (beta-1 antagonist) and propranolol (beta-1/2 antagonist) decreased REE by 6% (77 kcal/day) and 4% (48 kcal/day), respectively, and this was associated with decreased heart rate, oxygen consumption, and CO_2_ expiration [47]. In addition to increased heart rate, changes in energy substrate availability and energy production efficiency may also contribute to increased energy utilization in cardiac tissue. Different types of cancer are associated with different cardiac glucose uptake rates measured by ^18^F-FDG positron emission tomography CT scans [60], which changes the ratio of glucose to fatty acids being oxidized, and subsequently the oxygen requirements in cardiac tissue. A summary of these studies identifying metabolic changes is found in Table 1.

Metabolic aberrations leading to increased energy expenditure [18,20,94] and increased futile cycling [25,26,27] are key components of cachexia-induced skeletal muscle wasting and may therefore also have a role in cardiac atrophy and dysfunction. Although little is known clinically in this regard, pre-clinical work is beginning to uncover evidence of metabolic dysfunction in the cachectic heart.

### 4.2. Mechanistic Contributors to Cardiac Metabolic Changes

**Figure 2:**
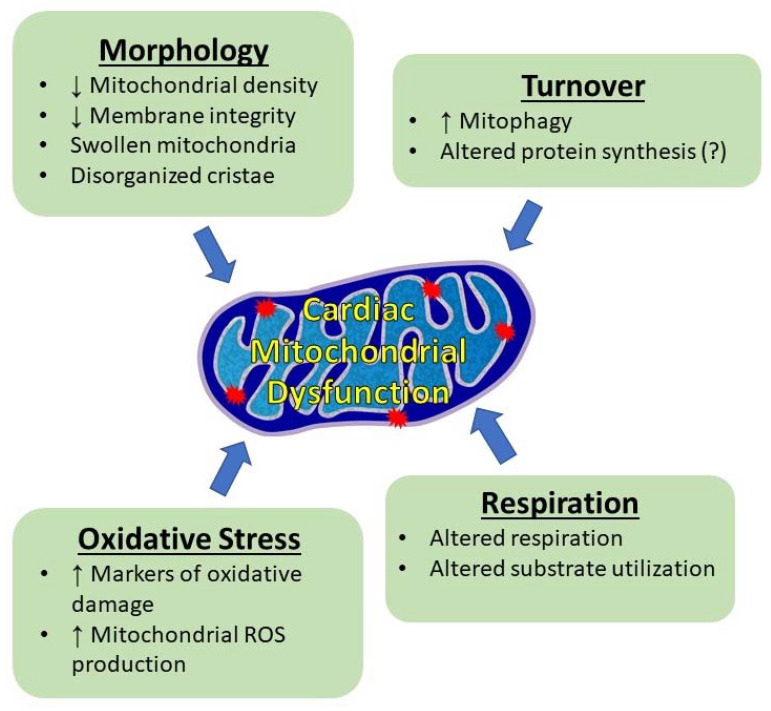
Summary of cardiac mitochondrial changes in cancer cachexia that may contribute to impaired cardiac function.

The healthy heart is a metabolically demanding organ that relies primarily on mitochondrial metabolism to provide the necessary ATP for contraction, relaxation, and the maintenance of resting membrane potential. It is estimated that the heart consumes approximately six kilograms of ATP per day [96]. To support this large metabolic demand, mitochondria make up between 30–40% of the volume of cardiac myocytes and produce approximately 95% of the ATP [97]. Beyond energy production, mitochondria also play a pivotal role in regulating cellular redox balance and are a primary site of reactive oxygen species production [98]. Therefore, this section on mechanisms of metabolic remodeling will highlight cancer-induced mitochondrial alterations in key mitochondrial-related measurements from animal models. Figure 2 presents an overview of findings related to mitochondrial health and function.

#### 4.2.1. Substrate Utilization

It is well established that the healthy, normal heart preferentially uses fatty acid β-oxidation over carbohydrate sources (i.e., glucose and lactate) for ATP production. Isotope tracer studies estimated that ∼60–90% of the acetyl-CoA comes from β-oxidation of fatty acids [99] and 10–40% comes from the oxidation of pyruvate in the form of glucose or lactate [99]. To date, the data on the effect of cancer on cardiac substrate utilization are equivocal. An early study by Drott et al. demonstrated that hearts from tumor-bearing rats perfused in an ex vivo working heart preparation had decreased glucose uptake compared to fed and fasted control rats, which suggests either decreased capacity for glucose uptake into cells or a lower rate of glucose oxidation [100]. Addition of palmitate to the media of a cell culture model of cachexia caused increased maximal oxygen consumption and upregulation of several genes related to lipid metabolism [63], lending support to the ex vivo heart findings from Drott et al. In contrast, Lee et al. indirectly estimated oxidative vs. glycolytic flux using two-photon excitation fluorescence to assess the redox ratio between FAD and NADH in hearts from cachectic mice [101]. A lower redox ratio was found in this study, indicating decreased utilization of mitochondrial oxidative metabolism and a greater reliance on glycolysis. In support of this finding, proteomic profiling identified downregulation of several mitochondrial electron transport chain proteins [82]. One potential explanation for this finding was the potential for mild hypoxia. HIF1α protein, a transcription factor activated by hypoxia, was significantly elevated in hearts from tumor-bearing animals [101,102]. These studies used HIF1α as a marker of hypoxia, which, in the context of decreased venous oxygen concentration [102], increased skeletal muscle HIF1α [102], and decreased skeletal muscle capillary density [103], suggests impaired oxygen delivery to tissues. Together, these studies implicate hypoxia as a potential contributor to cachexia, which would likely alter substrate utilization.

Unfortunately, substrate utilization in cardiac muscle cannot be inferred by comparison to skeletal muscle because their preferred substrate and regulation are different. For example, cancer-induced limb muscle wasting has been associated with insulin resistance [104,105,106], inhibition of glucose oxidation [107], and excessive fatty acid oxidation [108,109]. Moreover, there is a strong possibility that experimental results are model-dependent. Thackeray et al. [110] compared glucose metabolism in hearts of C26 and B16F10 tumor mice using PET scans for glucose uptake. In the advanced stage, cachectic hearts of C26 mice had elevated glucose uptake, consistent with the findings of Lee et al. [101]. On the other hand, B16F10 mice had lower glucose uptake [110], supporting the discovery of decreased glucose uptake found in ex vivo hearts by Drott et al. [100]. Therefore, further work is necessary to clarify the use of substrates and regulation of oxidative metabolism in the heart, especially in light of suggestions to use pharmacological inhibitors of fatty acid metabolism to treat/prevent skeletal muscle wasting in cachexia [111].

#### 4.2.2. Mitochondrial Morphology

One potential mechanism by which oxidative metabolism may be disrupted by cachexia is through changes in mitochondrial morphology. Electron microscopy images of hearts from multiple cancer cachexia mouse models reveal lower mitochondrial density, randomly dispersed and swollen mitochondria, impaired membrane integrity, and disorganized cristae [67,69,82,84]. Although not systematically studied, we speculate these structural changes to the inner mitochondrial membrane and cristae structure may potentially reduce oxidative phosphorylation capacity [112]. As is well-known, the cristae contain the electron transport chain (ETC) complexes. Currently, it is thought that the distance between these proteins and the cristae dramatically alters the rate of electron transfer through the ETC, and thus the rate of ATP production. For example, in highly oxidative tissue such as the heart, the cristae are closely stacked and take up most of the mitochondrial volume [113]. In less metabolically active tissue, such as the liver, the cristae are further apart [112]. Indeed, isolated mitochondria show condensed cristae when actively respiring, with cristae becoming more spread out in the absence of substrate [114].

#### 4.2.3. Mitochondrial Protein Synthesis and Mitophagy

In addition to changes in cristae structure, the protein content of the ETC complexes may also be reduced [64,69]. Like myocyte structural proteins, mitochondrial protein content is determined by the combination of protein synthesis through translation initiation and protein degradation through mitochondrial-specific autophagy (mitophagy). Several groups have demonstrated reduced cardiac protein synthesis [69,93]. It follows that since most mitochondrial proteins are nuclear encoded, reduced cellular protein synthesis would impact the mitochondrial fraction in a similar manner. Contrary to this thought, Lee et al. demonstrated elevated expression of mitochondrial biogenesis proteins (TFAM and TACO1) and mitochondrial-specific translation initiation factor proteins mtIF2 and mtIF3 in LLC tumor-bearing mice [101]. However, this did not lead to differences in mitochondrial protein synthesis in this study or in a recent study by Brown et al. [115]. Unfortunately, the ETC content does not resolve the different findings in these studies. Lee et al. demonstrated increases in ETC complex II and III, while Berent et al. [69] and Fernandes et al. [64] showed decreases in some of the complexes. These findings suggest that mitochondrial protein synthesis may be variable, and reduced mitochondrial volume [116] may be regulated by protein degradation more than protein synthesis.

The aforementioned structural changes and loss of membrane potential [116] in cachectic hearts would suggest that elevated degradation of mitochondria is required to maintain a healthy mitochondria population. Mitophagy, the selective form of macroautophagy (commonly referred to as autophagy), is capable of removing mitochondria. Unlike proteins in the cell that can be degraded by calpain or the UPS [117], the large organelle structure of mitochondria requires a process that can degrade lipids, protein, and DNA. This process is accomplished through the targeting of mitochondria and directed formation of a double membrane autophagosome, fusion with a lysosome, and degradation by the cathepsin family of proteases (reviewed in [118,119]). The majority of the autophagosome is consumed in the process of mitochondrial degradation and is not recycled, which makes accurate measurement of autophagy in frozen tissue samples challenging. Despite this, there are several markers which have been consistently associated with increased autophagy and commonly measured in the field. One such marker of autophagy is the ratio of microtubule-associated protein light chain 3 (LC3) II to LC3I (LC3II/LC3I) [120]. Several studies demonstrate an increase in the LC3 ratio in hearts of tumor-bearing animals [68,72,84,110,121]. These findings suggest that autophagy is increased in cardiac myocytes during cachexia and may lead to increased rates of protein degradation of mitochondria.

One of the more robust findings in cachexia literature is the increase in expression of BNIP3, a protein that is associated with the mitochondrial membrane and plays an important role in targeting degenerated mitochondria for mitophagy. A total of six papers found elevated BNIP3 mRNA or protein expression in cachectic hearts [62,64,68,72,121,122]. Only one study did not show an increase in BNIP3, and interestingly this study did not show cardiac atrophy or contractile dysfunction [123]. These findings are supported by electron microscopy images showing a higher number of double-membraned autophagic vacuoles in late-stage atrophic hearts [84]. A question that these data pose is whether mitophagy is beneficial or damaging to the heart? Is it specifically targeting damaged mitochondria in an attempt to make the overall population of mitochondria in the atrophied heart healthier? Or is the degradation non-specific and contributing to disorganized mitochondrial morphology and leading to mitochondrial dysfunction?

#### 4.2.4. Mitochondrial Respiration

Mitochondrial function is commonly defined by either respiration or reactive oxygen species production (ROS). First, mitochondrial respiration is estimated by oxygen consumption using modified definitions from Chance and Williams [124]. Briefly, carbon providing substrates (i.e., malate, pyruvate, succinate, glutamate, or palmitoylcarnitine) are added to isolated mitochondria or permeabilized fibers. Then, ADP is added as a substrate to stimulate a near maximal rate of respiration, classically defined as state 3 respiration. The second common measurement is state 4 respiration, which is either measured following ADP depletion or by the addition of oligomycin, an inhibitor of ATP synthase activity. One common interpretation of these data is to look at the respiratory control ratio (RCR) that is calculated by dividing state 3 by state 4. In theory, a healthy mitochondrion would have a high rate of respiration when stimulated by ADP and a low rate of respiration when ATP synthase is inhibited, which would create a high ratio of state 3 to state 4. Note that there are a variety of protocols for specific evaluation of substrates and electron transport chain activity covered in depth by Gnagier [125].

Smuder et al. reported impaired mitochondrial function in permeabilized cardiac myocytes from animals with C26 tumors [68]. These data demonstrated that cachectic hearts had lower state 3 respiration, higher state 4 respiration, and therefore a lower RCR compared to control animals. To our knowledge, this is the only study that has measured these parameters in an isolated ex vivo model. The other studies that have evaluated mitochondrial respiration have done so by using the previously mentioned method with cardiac myocytes cultured in conditioned medium from tumor cell culture. The conditioned medium was used to model the inflammatory cytokines released from the tumor that likely significantly contribute to cardiac atrophy. Berent et al. [69] did not find a difference in respiration in these cells in either state 3 or state 4 (listed as basal and leak, respectively), but did find a lower maximal rate of respiration, likely caused by the measured differences in ETC complexes. In a similar model, Nukaga et al. [116] added ascites from tumor animals in the cell culture media that lead to a reduced change in state 3 (basal), but no change in state 4 (leak). Although not calculated, these numbers would lead to a decreased RCR. Lee et al. also used this model and showed similar findings to Berent et al. [69], where there were no changes in basal or leak, but a lower maximal respiration capacity [101]. In contrast, Shäfer et al. demonstrated higher basal and leak respiration in cultured myocytes [63]. It is important to note that all these cell culture measurements were collected using the mitochondrial stress kit by Seahorse Bioscience, and there is still a debate on the interpretation and/or physiological relevance of these markers [126]. Additionally, all the cell culture studies were conducted with either immortalized cell lines (AC16 and H9c2) or primary neonatal rat cardiac myocytes, which have different characteristics compared to primary adult cardiac myocytes. Furthermore, cells were not paced in culture which drastically changes metabolic demand compared to the in vivo environment. Further studies using paced, adult cardiac myocytes may clarify discrepancies between studies.

#### 4.2.5. Oxidative Stress

In addition to alterations in mitochondrial respiration, the mitochondria can also contribute to the production of ROS, leading to oxidative stress that has been strongly associated with cachexia [127]. In skeletal muscle, mitochondria ROS production is increased before overt signs of cachexia such as muscle atrophy or changes in body weight occur [24,128]. Oxidative stress affects a multitude of pathways that may, in part, lead to the cardiac atrophy observed in cancer cachexia. Indeed, mitochondrial ROS can increase protein degradation, reduce protein synthesis, and reduce mitochondrial respiration [117].

Direct, in vivo measurement of ROS is technically difficult and therefore is commonly estimated through the measurement of markers of oxidative stress or in ex vivo approaches that use fluorogenic probes [129]. Markers of oxidative stress are reviewed in detail by Frijhoff et al. [130], but in general these markers measure stable by-products formed under redox stress. Examples include protein carbonyls formed through the oxidative cleavage of protein backbones, lipid oxidation products generated by H-atom abstraction, and DNA oxidation. Relatively limited evidence supports the notion that cancer leads to elevated oxidative damage to DNA through the measurement of deoxyguanosine (8-OHdG) [131,132]. Furthermore, TBARS, a marker of lipid peroxidation, was increased with no difference in protein carbonyl formation [133].

Two studies have used fluorogenic probes to provide high sensitivity and specificity real-time measurements for ROS. Mitochondrial hydrogen peroxide production was higher in hearts from tumor-bearing animals compared to control animals [68]. The use of a mitochondrial targeted antioxidant (SS-31) was able to prevent oxidative stress and restore function. Similarly, a mitochondrial-specific fluorogenic dye (MitoSOX) in cardiac myocytes cultured with media from cancer cells demonstrated a higher mitochondrial superoxide production [101]. These data are supported by unbiased RNA sequencing and pathway analysis data that revealed many differentially expressed genes related to oxidative stress [134]. In general, the volume of data is not strong enough to definitively state oxidative stress is present in cachectic hearts. However, the few studies that exist support the notion that cardiac muscle experiences oxidative stress in response to tumors, and the mitochondria are likely a significant source of ROS.

Whether or not oxidative stress is a cause of atrophy has long been debated in skeletal muscle atrophy models [135]. This mechanism has not been systematically studied in heart muscle. However, the best evidence is the indirect data using exogenous antioxidants to reduce oxidative stress. To date, four studies have been completed using supplements that have antioxidant properties in cells. Resveratrol, a polyphenol with strong ROS scavenging properties [136], prevented cardiac atrophy and dysfunction in the C26 mouse model [137]. Oxypurinol, which acts as an antioxidant by inhibiting xanthine oxidase ROS production, provided some protection against cardiac atrophy and contractile dysfunction in the Yoshida AH-130 hepatoma cancer cachexia model [138]. Neither of these studies measured markers of oxidative stress, sites of ROS production, or antioxidant capacity, which limits the ability to attribute these beneficial effects to the antioxidant properties. In cachexia cell culture, the addition of MitoTemo, a mitochondrial-specific antioxidant, was able to prevent increased superoxide concentrations and improve mitochondrial function and cell viability to a hypoxia challenge assay [101]. The mitochondrial-specific antioxidant SS-31 prevented increases in mitochondrial ROS production, and improved mitochondrial function and cardiac function, but did not demonstrate robust protection against cardiac atrophy [68].

#### 4.2.6. Future Research

There are several questions that remain to be answered about the mechanisms of altered bioenergetics and metabolism in cardiac cachexia. As the data show, substrate selection by the heart needs to be further explored and considered across a variety of models, especially in light of the opposite findings between models in the Thackeray study [110]. Translating these findings may lead to nutritional and pharmacological interventions to improve cardiac energy production and metabolism in cancer cachexia.

Mitochondrial physiology appears to play a prominent role in cardiac cachexia and may be a potential therapeutic target. To date, the field lacks well-controlled studies using genetic models to isolate specific pathways. Treatments with antioxidants and inhibitors have demonstrated potential, however, care should be taken to understand if these molecules increase tumor burden. Of note, none of the studies presented here [68,137,138] reported increased tumor growth. However, many exogenous supplements [139,140,141] and even tissue-specific genetic manipulation [142] can increase tumor growth rate, making tumor growth an important consideration moving forward.

## 5. Conclusions

**Figure 3:**
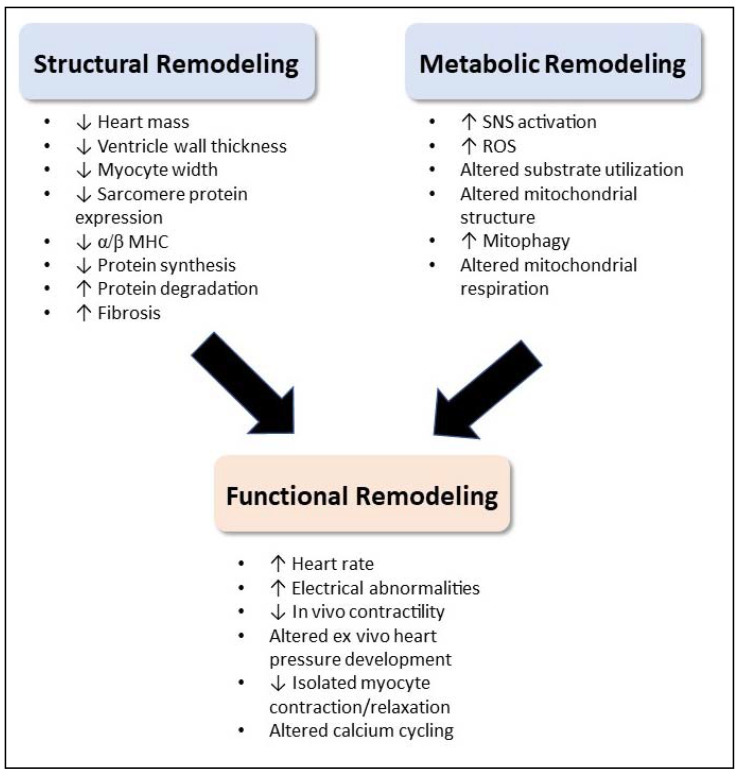
Overview of cardiac alterations in cancer-induced cachexia. Both structural and metabolic remodeling likely contribute to decreased function.

The development and progression of cancer-induced cachexia involves a complex interplay between tumor- and host-derived factors that involves multiple tissues and organs [34]. One of the organs involved in this syndrome is the heart. Although atrophy of the cardiac muscle in cachectic patients was observed decades ago [78], we are just beginning to understand the underlying mechanisms of cachexia-induced cardiomyopathy. Increasing evidence of cardiac functional, structural, and metabolic changes in patients with cancer cachexia suggests the heart is significantly affected by, and also contributes to, cachexia pathophysiology, making the heart a vital area of cachexia research. Figure 3 presents an overall summary of the findings.

Cachexia-induced cardiomyopathy has both similarities and differences compared with other types of heart failure, which affects the clinical management of this condition. Both clinical and pre-clinical studies have identified impaired systolic function [54,67], with some work also identifying impaired diastolic function [48,74]. Decreased calcium cycling [74], increased fibrotic development [48,67], and changes in myosin isoform expression [66,67] may all contribute to functional deficits and are all common to multiple cardiomyopathy etiologies. However, the atrophic structural changes are in stark contrast to either the hypertrophic or dilated phenotypes characteristic of many other cardiomyopathies [35,143,144]. Decreased total expression of sarcomeric proteins [66], decreased cardiac myocyte width in the absence of increased length [74], and thinning of the ventricular walls [48,66] suggests an imbalance between proteolysis and protein synthesis, similar to cachectic skeletal muscle. Atrophy may be a contributor to the overall decrease in force production in the cachectic heart [52], although additional structural and morphological changes such as t-tubule remodeling occurring in conjunction with decreased sarcomeric protein expression may also contribute and should be investigated [145]. Furthermore, similar to other forms of heart failure, significant alterations in whole body and cardiac metabolism may hinder energy-requiring functions in the heart, such as maintenance of electrochemical gradients and intracellular calcium concentrations, and cross-bridge cycling. Considerable evidence exists for cachexia-induced skeletal muscle mitochondrial dysfunction, with increasing evidence showing mitochondrial abnormalities in the heart as well [68,101]. As the heart has a high metabolic demand, contributing significantly to basal metabolic rate [95], decreased energy production by cardiac mitochondria may contribute to both functional deficits and atrophy of the heart muscle.

Published literature related to the cardiac effects of cancer cachexia reviewed in this paper identifies several consistent findings. First, in vivo systolic dysfunction (decreased contractility) was found in most pre-clinical studies undertaking echocardiographic measurements [48,65,67], and this was corroborated by limited clinical data also examining cardiac function in cachectic patients [46,54,56]. Second, cardiac atrophy was a consistent finding in many pre-clinical models [48,67,71,81] as well as some studies in humans [48,57]. Finally, evidence of autophagy was significantly and consistently increased in numerous pre-clinical models [68,72,121]. These findings provide important inroads into our understanding of cachexia-induced cardiomyopathy. However, many questions remain related to the mechanistic underpinnings of cachexia-induced cardiomyopathy. Specifically, there are several areas in which existing studies present contradictory findings. Related to cardiac function, some evidence suggests that isolated heart and cardiac myocyte functional deficits underlie in vivo dysfunction [74]; however, not all studies are in agreement [76]. Related to metabolism and mitochondrial function, further work is needed to understand changes in substrate utilization, mitochondrial respiration, and mitochondrial protein synthesis. In addition to understanding contradictory findings in existing literature, there are other areas of research that should be considered. First, identification of cardiac changes early in cachexia pathogenesis will enable identification of biomarkers to predict cardiomyopathy risk, as well as enable targeted therapeutic development [24]. Second, understanding the reversibility of cachexia-induced cardiac dysfunction in patients who are living with stable disease or are in remission will be vital to development of long-term management strategies.

Research progress surrounding cachexia-induced cardiomyopathy is not without challenges. Clinically, identification of cachexia-specific effects on the heart is difficult to ascertain. The type and stage of the tumor, cancer therapies, and pre-existing cardiovascular disease are all factors that complicate the interpretation of clinical data. Although animal models have similarities with the human condition, the high tumor burden, young age, non-metastatic disease, and rapidity of cachexia development in the most commonly used models pose challenges to clinical translatability [146,147]. Finally, mice have differences in cardiac structure and function compared to humans [148,149], necessitating care in the interpretation and extrapolation of pre-clinical findings to humans. Nevertheless, the parallel nature of clinical and pre-clinical research in cachexia-induced cardiomyopathy has provided important inroads into understanding this complex condition.

In conclusion, cachexia-induced cardiomyopathy is characterized by impaired in vivo systolic function, significant atrophy of the cardiac muscle, and evidence of energy production and mitochondrial functional deficits. Because cardiac insufficiency can lead to muscle wasting independent of cancer [35], cardiac dysfunction in cachexia may contribute to cachexia pathophysiology. Indeed, many symptoms, including fatigue, exercise intolerance, and dyspnea, are shared between cancer cachexia and heart failure [36]. Therefore, understanding the effects of cancer cachexia on the heart is imperative for optimal clinical management and improved treatment options for patients with cachexia-induced cardiomyopathy.

## Figures and Tables

**Table 1:** Overview of Clinical Studies Related to Cardiac Alterations in Cancer and Cancer-Induced Cachexia.

	Patients (Sample Size)	Study Design	Major Findings
**Functional Remodeling**			
Anker, Ebner, Hildebrandt, et al. 2016 [45]	NSCLC, pancreatic, CRC (145)	Prospective, longitudinal	HR > 75 bpm predicted for mortality
Anker, von Haehling, Coats, et al. 2021 [53]	NSCLC, pancreatic, CRC (120)	Prospective, longitudinal	- ↑ NSVT vs. control - NSVT & PVC predicted for mortality
Cramer, Hildebrandt, Kung, et al. 2014 [46]	CRC (50)	Prospective, single timepoint	↑ HR, ↓ HRV, ↓ LVEF vs. control
Kazemi-Bajestani, Becher, Butts, et al. 2019 [54]	NSCLC (50)	Prospective, longitudinal	↓ LVEF, ↓ GLS at 4-month follow-up
Kazemi-Bajestani, Becher, Butts, et al. 2019 [56]	NSCLC (70)	Prospective, single timepoint	↓ LVEF (<50%) incidence is higher in cachectic vs. non-cachectic patients
Lee, Park, Lim, et al. 2016 [44]	Breast cancer (4786)	Retrospective, longitudinal	↑ HR predicted for mortality
**Structural Remodeling**			
Barkhudaryan, Scherbakov, Springer, et al. 2017 [57]	Lung, pancreatic, GI cancer (177)	Retrospective, single timepoint	↓ Heart weight in cachectic vs. non-cachectic patients
Cai, Mao, Yang, et al. 2020 [58]	Pancreatic cancer (98)	Retrospective, longitudinal	↓ LVMA, LVMRA associated with mortality
Kazemi-Bajestani, Becher, Butts, et al. 2019 [54]	NSCLC (50)	Prospective, longitudinal	Cardiac atrophy associated with ↑ DLT, ↓ treatment response, ↓ physical functioning, ↑ mortality
Springer, Tschirner, Haghikia, et al. 2014 [48]	NSCLC, pancreatic, GI cancer (12 cancer, 14 cancer cachexia)	Post-mortem	Cachexia associated with ↓ heart mass, ↓ LVWT
**Metabolic Remodeling**			
Hyltander, Körner, Lundholm, 1993 [59]	Weight-losing cancer patients (60)	Randomized, controlled trial	SNS activation is a main driver of increased REE
Hyltander, Daneryd, Sandström, et al. 2000 [47]	Weight-losing cancer patients (10)	Randomized, crossover	SNS attenuation via β-blockers caused ↓ REE, ↓ HR, ↓ O2 uptake
Heckmann, Totakhel, Finke, et al. 2019 [60]	Hodgkin’s lymphoma, non-Hodgkin’s lymphoma, non-lymphatic cancer (337)	Retrospective, single timepoint	- Hodgkin’s lymphoma associated with ↑ cardiac glucose uptake - Chemotherapy associated with ↓ cardiac glucose uptake

Colorectal cancer (CRC); dose-limiting toxicity (DLT); global longitudinal strain (GLS), heart rate (HR); heart rate variability (HRV); left ventricular ejection fraction (LVEF); left ventricular muscle area (LVMA); left ventricular muscle radiation attenuation (LVMRA); left ventricle wall thickness (LVWT); non-small cell lung cancer (NSCLC); non-sustained ventricular tachycardia (NSVT); premature ventricular contractions (PVC); resting energy expenditure (REE); sympathetic nervous system (SNS).
